# Safe and successful CAR T-cell therapy targeting BCMA in a multiple myeloma patient requiring hemodialysis

**DOI:** 10.1007/s00277-023-05163-z

**Published:** 2023-03-17

**Authors:** Ralph Wäsch, Tim Strüssmann, Claudia Wehr, Reinhard Marks, Phillip T. Meyer, Gerd Walz, Monika Engelhardt

**Affiliations:** 1 Department of Hematology, Oncology and Stem Cell Transplantation, Medical Center - University of Freiburg, Faculty of Medicine, University of Freiburg, Hugstetterstrasse 55, 79106 Freiburg, Germany; 2 Department of Nuclear Medicine, Medical Center - University of Freiburg and Faculty of Medicine, University of Freiburg, Freiburg, Germany; 3 Department of Medicine IV, University Freiburg Medical Center, Faculty of Medicine, University of Freiburg, Freiburg, Germany

Dear Editor,

We report here on a patient with multiple myeloma (MM) on hemodialysis receiving a chimeric antigen receptor (CAR) T-cell therapy targeting B-cell maturation antigen (BCMA) [1, 2]. Patients with severe renal insufficiency (RI) or receiving hemodialysis have typically been excluded from clinical trials. Thus, data for these MM patients are scarce, although now exist for patients with diffuse large B-cell lymphoma (DLBCL) [3, 4].

**Fig. 1:**
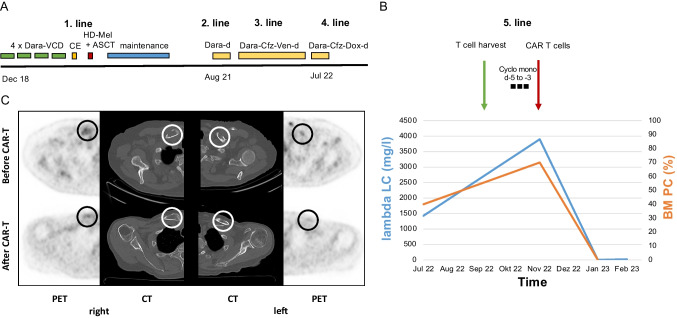
**A** Therapeutic timeline before CAR T cell therapy. **B** Rapid response to CAR T cell therapy indicated by normalization of lambda LCs and bone marrow infiltration rate. **C** Regression of metabolic activity of exemplary PET-positive myeloma lesions and remineralization of osteolyses in both clavicles following CAR T cell therapy

Our 74-year-old patient suffered from lambda light chain (LC) MM, which was diagnosed in 12/2018 with standard-risk cytogenetics (t(11;14)). At initial diagnosis (ID), he presented with 3/4 CRAB criteria: acute renal failure with creatinine 8.65 mg/dl (GFR 6 ml/min/1.73qm), anemia of 10.8 g/dl and osteolyses. He had a bone marrow infiltration rate of 80% plasma cells and serum lambda LCs of 9450 mg/l. A renal biopsy revealed cast nephropathy. He received plasmapheresis and induction therapy with daratumumab-bortezomib-lenalidomide-dexamethasone (Dara-VCD) leading to vgPR after one cycle. He was treated with a total of four cycles followed by stem cell mobilization with cyclophosphamide-etoposide (CE) and granulocyte-colony-stimulating factor (G-CSF) while receiving hemodialysis. High-dose chemotherapy with melphalan 140 mg/m^2^ and autologous stem cell transplantation (auto-SCT) was performed leading to CR and termination of hemodialysis with creatinine values around 4–5 mg/dl and sufficient renal function. Maintenance therapy with VD was stopped after one cycle due to polyneuropathy (PNP) and continued with pomalidomide 1 mg due to his RI for 12 months (Fig. 1A).

2 ½ years after ID in 08/2021, the first lambda LC-progression occurred, and the patient received second-line therapy with Dara-dexamethasone (d) leading to stable disease after two cycles. Due to insufficient response (> PR), third-line therapy with Dara-carfilzomib (cfz)-venetoclax (ven)-d was initiated, since the patient harbored t(11;14) (Fig. 1A). Unfortunately, the disease continued to slowly progress and in 07/2022, a rapid increase of lambda LCs reoccurred. Bone marrow biopsy showed a plasma cell infiltration rate of 40%. After approval of reimbursement, a CAR T-cell therapy was planned. A successful T-cell harvest was performed in 09/22. Due to cytopenia, venetoclax was not resumed, and low-dose doxorubicin was added instead when the disease progressed further. In 11/2022, the bone marrow infiltration increased to 70% and lambda light chain to a maximum of 3900 mg/l. The patient went on hemodialysis again.

After lymphodepletion with cyclophosphamide 300 mg/m2 from day -5 to day -3, the patient received Idecabtagen-Vicleucel (Abecma®, ide-cel) [5, 6] (Fig. 1B). Lymphodepletion with fludarabine was omitted due to marginal renal function. Ide-cel was very well tolerated with cytokine release syndrome (CRS) grade 1 and no immune-effector cell-associated neurotoxicity syndrome (ICANS). Two months after CAR T-cell therapy the patient is in sCR with normalization of lambda light chains, negative immunofixation, and disappearance of clonal plasma cell infiltration in the bone marrow (Fig. 1B). Monitoring by [^18^F]FDG PET/CT demonstrated regression of metabolic activity of myeloma lesions with remineralization of osteolyses (Fig. 1C).

Our case illustrates the feasibility of CAR T-cell therapy in MM patients with end-stage renal disease on hemodialysis, thus warrants more MM patients not to be excluded from this valuable cell therapy approach.
